# Influences of Vitamin A on Vaccine Immunogenicity and Efficacy

**DOI:** 10.3389/fimmu.2019.01576

**Published:** 2019-07-17

**Authors:** Rhiannon R. Penkert, Hannah M. Rowe, Sherri L. Surman, Robert E. Sealy, Jason Rosch, Julia L. Hurwitz

**Affiliations:** ^1^Department of Infectious Diseases, St. Jude Children's Research Hospital, Memphis, TN, United States; ^2^Department of Microbiology, Immunology and Biochemistry, University of Tennessee Health Science Center, Memphis, TN, United States

**Keywords:** vitamin A, pneumococcus, supplementation, vaccines, immune response

## Abstract

Vitamin A deficiencies and insufficiencies are widespread in developing countries, and may be gaining prevalence in industrialized nations. To combat vitamin A deficiency (VAD), the World Health Organization (WHO) recommends high-dose vitamin A supplementation (VAS) in children 6–59 months of age in locations where VAD is endemic. This practice has significantly reduced all-cause death and diarrhea-related mortalities in children, and may have in some cases improved immune responses toward pediatric vaccines. However, VAS studies have yielded conflicting results, perhaps due to influences of baseline vitamin A levels on VAS efficacy, and due to cross-regulation between vitamin A and related nuclear hormones. Here we provide a brief review of previous pre-clinical and clinical data, showing how VAD and VAS affect immune responses, vaccines, and infectious diseases. We additionally present new results from a VAD mouse model. We found that when VAS was administered to VAD mice at the time of vaccination with a pneumococcal vaccine (Prevnar-13), pneumococcus (T4)-specific antibodies were significantly improved. Preliminary data further showed that after challenge with *Streptococcus pneumoniae*, all mice that had received VAS at the time of vaccination survived. This was a significant improvement compared to vaccination without VAS. Data encourage renewed attention to vitamin A levels, both in developed and developing countries, to assist interpretation of data from vaccine research and to improve the success of vaccine programs.

## Introduction

Vitamin A deficiency (VAD) adversely affects children and adults worldwide. Today, the World Health Organization (WHO) estimates that 250 million preschool children suffer from VAD, with the highest frequencies among low-income areas of Africa and South-East Asia [http://www.who.int (accessed March 01, 2019)]. Infectious diseases, particularly respiratory and diarrheal diseases, occur at increased frequencies (at least 2:1) among populations with VAD compared to vitamin-replete populations (1).

The global burden of VAD and its effects on populations in developing countries are well known, but much less well appreciated are incidences of VAD and vitamin insufficiencies in the developed world (2–5). In Memphis, TN, we tested influenza virus-infected children and their household contacts for retinol binding protein (RBP) as a surrogate for vitamin A (6), and found that 13 of 21 individuals were either vitamin A insufficient or deficient (5). We found that both infected and uninfected study participants exhibited low RBP levels. Low RBP can be a consequence of illness (7), but also reflects conditions of malnutrition when individuals in low-income families have limited access to nutrient-rich foods (8–10). Whereas, infants in the United States may receive government-funded, vitamin-fortified formulas, comparable support is not given to older children (11). Instead, diets for older children and adults may be calorie-dense and nutrient-poor. As is the case in developing countries, vitamin insufficiencies and deficiencies in the developed world correlate with weakened immune responses and poor outcomes upon hospitalization for infectious disease (2, 4). Unlike the situation in the developing world, individuals in the United States are usually assumed to be vitamin A-replete. Malnutrition may therefore go unnoticed.

### Vitamin A Requirements, Metabolism, and Trafficking

Vitamin A is acquired from the diet in the form of retinoids (preformed vitamin A) or carotenoids (provitamin A). Retinoids include retinol or retinyl esters from animal sources, whereas carotenoids include beta-carotenes from plants. The recommended daily allowance (RDA) for vitamin A is dependent on age and sex. The Office of Dietary supplements (ODS) at the National Institutes of Health (NIH) currently recommends an RDA ranging from 300 to 600 μg retinoic acid equivalents (RAE) for young children. For individuals aged ≥14 years, RDAs are 700 μg RAE for non-pregnant females and 900 μg RAE for males (1 IU retinol = 0.3 microgram RAE) [https://ods.od.nih.gov (accessed March 01, 2019)]. A blood level of <0.7 μM retinol is considered vitamin “deficient” or “inadequate,” and levels between 0.7 and 1.05 μM retinol are considered “insufficient” or “marginal” for some biological functions (12).

Vitamin A is generally stored in the liver as esters, but can also be found in extra-hepatic sites such as lung, intestine, kidney, and adipose tissue (13, 14). Retinol is the most common vitamin A metabolite in the blood and typically circulates in a complex bound to RBP with a 1:1 molar ratio. Retinol-bound RBP (holo-RBP) is, in turn, often bound to transthyretin, a common serum transport protein (15, 16). Retinoids can also be transported by chylomicrons or chylomicron remnants in lymph and blood (14, 17). Intracellularly, retinol is converted by retinol dehydrogenases (RDH, ubiquitous enzymes) to retinal, and then by retinaldehyde dehydrogenases (RALDH, e.g., ALDH1A) in select tissues to retinoic acid (RA) (18–21). RA is the vitamin A metabolite best known for its ability to regulate innate and adaptive immune cell function, proliferation, and survival. Importantly, metabolism and trafficking of vitamin A, and consequent effects on the immune system, can be influenced by genetic backgrounds, diets, conditions of mal-adsorption, and obesity (22). In the case of obesity, animal experiments suggest that vitamin A may be deficient in tissues such as the lung, even when levels in blood appear to be replete (23).

### Regulatory Functions of Vitamin A

Virtually every mammalian cell, including epithelial and immune cells, is affected by vitamin A (7, 24–34). Vitamin A is perhaps best known for its regulation of gene transcription. RA is a nuclear hormone that binds nuclear hormone receptors including the retinoic acid receptor (RAR) and the peroxisome proliferator-activated receptor (PPARβ/δ) (35, 36). Receptors, in turn, bind DNA and serve as transcription factors to enhance or inhibit gene expression (27). Multiple isoforms exist for RAR [e.g., RARα, β, and γ (37)], and each protein can bind to the retinoid X receptor (RXR) in a heterodimeric complex (27, 38).

Receptors are promiscuous in binding to their ligands and to DNA. The RAR-RXR heterodimer will often bind two half-site sequences, known as retinoic acid response elements (RAREs), separated by a short spacer in the DNA (27, 39–44). RAREs have a consensus sequence of 5′-(A/G)G(G/T)TCA-3′, though receptors can be bound to non-consensus DNA sites as well. The exact sequence and spacer length (typically zero to eight bases) can alter binding affinity. Additionally, receptors can bind indirectly to DNA by tethering to other DNA-bound factors. Cross-regulation between vitamin A and related nuclear hormones (e.g., vitamin D, thyroid hormone, or sex hormones) may occur, because nuclear hormone receptors can compete for binding to ligands, co-receptors, and DNA (27, 40, 45–48).

RAREs are found throughout the genome, often within gene promoters or enhancers. Notably, hotspots for RARE have been identified in switch sites of the immunoglobulin heavy chain locus, positions instrumental in class switch recombination (CSR) (49). The potential binding of nuclear hormone receptors to switch sites and regulatory elements in immunoglobulin and T cell receptor (TCR) loci predicts a direct mechanism by which vitamin A may modulate lymphocyte function (49–52).

Adding to the complexity of vitamin A functions are the extra-nuclear activities. Vitamins bind a complex array of escort proteins at the cell membrane and in extra-nuclear compartments. Each of these interactions can initiate or modulate cell signaling (53, 54).

### Vitamin A and Immune Activities *in vitro* and in Small Animals

Essentially all cells of the immune system including innate cells, B cells, and T cells, are affected by vitamin A (31, 55–59). Research animals with VAD generate poor antibody responses to many pathogens including parainfluenza virus and influenza virus (34, 59–61). VAS, when administered either orally or intranasally, can correct responses when given at the time of vaccination (33, 59, 60, 62).

*In vitro*, vitamin A has been shown to upregulate IgA production by B cells (18, 63, 64), and skew T cell phenotypes toward Treg rather than Th17 populations (65–70), but *in vivo*, outcomes are less predictable (71). For example, whereas VAD cells may yield poor Treg activities in a tissue culture setting indicating a predisposition for heightened immune responses (65–68), Tregs are found at equal or greater frequencies in tissues of VAD mice compared to controls following a respiratory virus infection (32). Furthermore, VAD mice exhibit relatively poor pathogen-specific T cell responses *in vivo*. In studies of influenza virus and parainfluenza virus infections, there are only weak virus-specific CD8+ T cell responses in the lower respiratory tract (LRT) of VAD mice (26). Responding CD8+ T cells in VAD and vitamin A+D deficient (VAD+VDD) mice express high levels of membrane CD103 (the αE subunit of αEβ7, an e-cadherin receptor). Possibly, the poor recruitment of CD8+ T cells to the LRT is because LRT tissues express relatively low levels of e-cadherin, and CD103+ cells home preferentially to other sites (26). When VAD+VDD mice receive VAS (with or without supplemental vitamin D), CD103 levels on virus-specific CD8+ T cells are reduced, and the percentages of CD4+ and CD8+ T cells in the LRT are improved (72). As another example of the complex influences of vitamin A on immune responses, we find that serum antibody isotype distributions differ between VAD and control animals, but patterns are dependent on the animal's background and sex (50). As a last example, some studies show that VAD biases the immune response toward a Th1 profile and that high levels of vitamin A bias the response toward a Th2 profile (68, 73, 74). Nonetheless, outcomes are again dependent on cell targets, environment, and activation state (25). Both Th1 and Th2 cytokine responses are evident in VAD mice, and VAD animals express higher levels of Th1 and Th2 cytokines compared to controls at late stages following a respiratory virus infection, presumably as a consequence of poor virus clearance (32).

Vitamin A additionally influences epithelial cells and innate immune cells associated with mucosal surfaces. Dendritic cells of the intestine and epithelial cells of the respiratory tract each express the ALDH1A enzymes required for conversion of retinaldehyde to the end-metabolite RA (18, 26, 29). These unique attributes of mucosal tissues help explain why VAS assists immune responses when applied either orally or intranasally (33, 62).

Due to the plethora of immune cell and barrier cell requirements for vitamin A, it is not surprising that VAD associates with poor immune responses to vaccines, and that VAS can reverse these weaknesses when given at the time of vaccination (33, 34, 59–62). One vaccine that deserves continued study in the context of VAD and VAS is Prevnar-13. It is estimated that worldwide pneumococcus kills close to 1 million children under the age of 5 each year (75, 76). Prevnar-13 can protect against these mortalities (77, 78), but the vaccine-induced immune response is not always protective. We suggest that attention to, and correction of, low vitamin levels in Prevnar-13 vaccine recipients may improve vaccine success. Previous studies have shown that VAD inhibits responses both to individual pneumococcus antigens and to Prevnear-13 in mice (59, 61, 79–81). Here, we extend findings to show that VAS improves the immunogenicity and protective capacity of Prevnar-13 in VAD and control animals.

## Materials and Methods

### Mice and Vaccinations

Experiments were reviewed and approved by the Institutional Animal Care and Use Committee (IACUC) at St. Jude Children's Research Hospital (St. Jude). St. Jude follows the standards of the Animal Welfare Act and the document entitled “Principles for the Use of Animals and Guide for the Care and Use of Laboratory Animals.”

To produce VAD mice, pregnant C57BL/6 (H2-b) mice were purchased from Jackson Laboratories (Bar harbor, ME). Mice were placed on either a control or VAD diet upon their arrival in the animal facility at St. Jude (days 4–5 gestation). VAD (cat. no. 5WA2, Test Diets) and control (cat. no. 5W9M) diets differed only in vitamin A content, containing either 0 or 15 IU/g vitamin A palmitate, respectively. Mothers and progeny remained on their assigned diets. Experiments were begun when progeny reached adulthood. These adult mice were vaccinated with 2 doses of Prevnar-13 (PCV, Wyeth Pharmaceuticals Inc.) with 3 weeks intervals. Vaccine was diluted 1:40 in PBS and 100 μL of PCV was administered intraperitoneally (IP). Immediately prior to each vaccination, mice received either 600 IU vitamin A (from Interplexus Inc., Kent, WA) or PBS by oral gavage (100 μL).

### Antibody Measurements by ELISA

Animals were bled 10–14 days after boosting. ELISA plates were coated with 50 μL/well of 5 μg/mL T4 polysaccharide (from American Type Culture Collection, ATCC, Manassas, VA) in PBS using an Integra Viaflo384 robot (Integra Biosciences, Hudson, NH) and incubated overnight at 4°C. Plates were then washed 3x with PBS using an Aquamax 4000 plate washer (Molecular Devices, San Jose, CA). Block was 1% BSA in PBS (200 μL/well) added robotically and incubated overnight at 4°C. Mouse serum samples were diluted 1:500 in dilution buffer (1% BSA + 0.05% Tween in PBS). Block was removed and samples were added to plates (50 μL/well) and incubated overnight at 4°C. Plates were then washed 3x with PBS +0.05% Tween using the plate washer. Developing antibodies were added robotically (100 μL/well). These were anti-mouse IgM (cat. no. 1020-04; Southern Biotech, Birmingham, AL), anti-mouse IgG1 (cat. no. 1070-04; Southern Biotech), or anti -mouse IgG3 (cat. no. 1100-04; Southern Biotech), each diluted 1:1000 in dilution buffer. Plates were incubated 1 h at room temperature and then washed 3x with PBS +0.05% Tween using the plate washer. Substrate (1 mg/mL of pNPP in diethanoloamine buffer; 100 μL/well) was added robotically to plates. Plates were developed for 5–15 min and read at 405 nm on a VersaMax Tunable Microplate Reader (Molecular Devices). Statistical comparisons were made using Mann Whitney tests and GraphPad Prism software (^*^p < 0.05, ^**^p < 0.01, ^***^p < 0.001).

### Challenges Post-vaccination

To prepare bacteria for challenge experiments, *S. pneumoniae* strain TIGR4 (serotype 4) was inoculated from a glycerol stock onto a Tryptic Soy Agar plate (GranCult, Millipore, Burlington, MA) supplemented with 3% sheep blood (Lampire Biological Laboratory, Pipersville, PA) and 20 μg/mL neomycin, and grown at 37°C, 5% CO_2_. After overnight growth, bacteria were directly inoculated into Todd Hewitt broth (Becton Dickinson, BD, Sparks, MD) supplemented with 0.2% yeast extract (BD) and grown until mid-log phase, OD_620_ = 0.4. Cells were washed in PBS prior to animal infections.

To challenge mice, 2 weeks after the vaccine boost, animals were sedated with 3% isoflurane. They were then inoculated intranasally with 5 × 10^5^ CFU *S. pneumoniae* in 100 μL PBS. To collect and titer lungs, 24 h after infections groups of animals were euthanized by CO_2_ asphyxiation and cervical dislocation. Lungs were removed, washed twice in ~1 mL of PBS and then placed in 0.5 mL PBS. Lungs were then pulverized with a mechanical tissue grinder. Following emulsification, lungs were spun for 5 min at 300 g to pellet debris. Supernatants from the lung homogenates were collected and serially diluted 1:10 in PBS five times. From each dilution, 10 μL were plated on a Tryptic Soy Agar plate (GranCult, Millipore) supplemented with 3% sheep blood (Lampire Biological Laboratory) and 20 μg/mL neomycin. Plates were incubated overnight at 37°C. Colonies were counted and Excel software was used to calculate titers. Separate groups of animals were infected as described above, monitored for signs of symptomatic infection, and euthanized when moribund.

## Results

**Figure 1:**
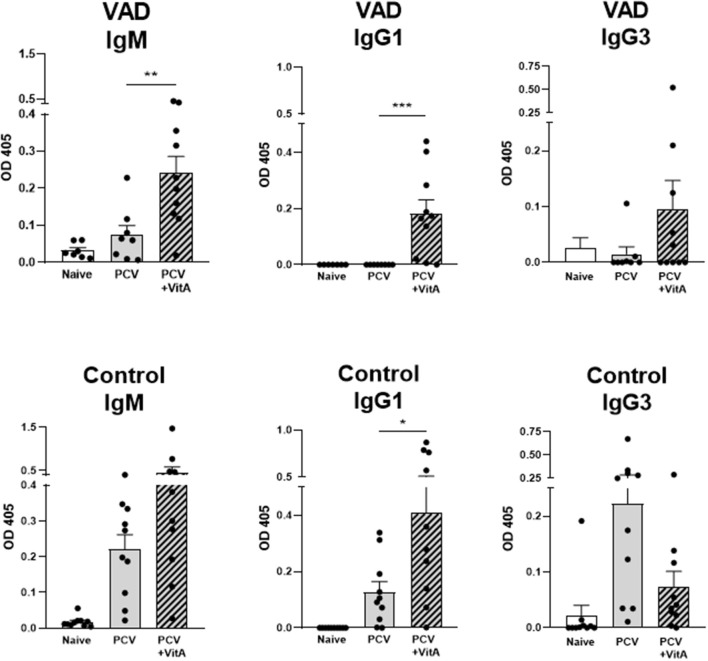
VAS and T4 polysaccharide-specific immune responses. Results from T4 ELISAs are shown for VAD (top row) and vitamin A-replete control (bottom row) mice. Separate ELISAs were conducted to measure T4-specific IgM IgG1, and IgG3 antibodies. Statistical comparisons were made using Mann Whitney tests and GraphPad Prism software (*p < 0.05, **p < 0.01, ***p < 0.001). IgM levels (for VAD mice), and IgG1 levels (for VAD and control mice), but not IgG3 levels, were significantly improved with VAS.

Vaccine studies were conducted with male and female mice (either VAD mice or vitamin-replete controls) that were given two successive IP immunizations, separated by 3 week intervals, with the Prevnar-13 vaccine. Mice received either 600 IU of vitamin A as retinyl palmitate by oral gavage or phosphate buffered saline (PBS) at the time of vaccination. Antibody responses were measured 10–14 days after the second vaccine dose. ELISAs were conducted to examine antibodies specific for the type 4 (T4) component of the vaccine. As shown in Figure 1, there was significant improvement of T4-specific antibodies, including IgM and IgG1 isotypes in VAD mice and IgG1 in control mice when VAS was used. IgG3 levels were not significantly changed. Results were reminiscent of previous studies in rats using bacterial antigens and retinol treatments (79–82).

**Figure 2:**
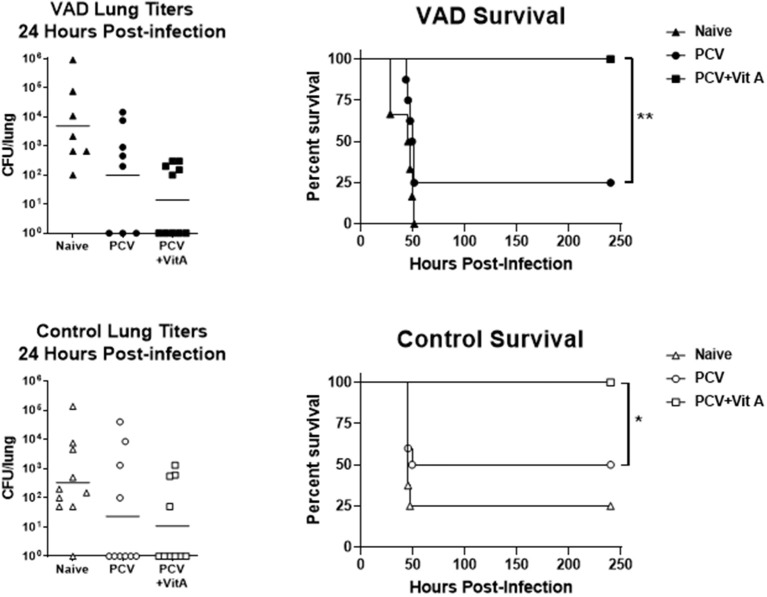
VAS with vaccination improves survival after challenge. Challenge results are shown for VAD (top row) and vitamin-replete control (bottom row) mice. CFU per lung were measured 24 h after challenge (left). In separate groups of mice, survival was monitored (right). Animals were sacrificed when moribund. Survival curves were compared using GraphPad Prism software (*p < 0.05, **p < 0.01).

In a preliminary set of experiments, vaccinated animals were also challenged with a high-dose (5 × 10^5^ colony forming units, CFU) of pneumococcus (*Streptococcus pneumoniae* strain TIGR4 [serotype 4]). At this dose, >90% of unvaccinated VAD and vitamin-replete control mice developed an infection, and the dose was 100% lethal in unvaccinated VAD animals (Figure 2). After 24 h, groups of 8–10 mice were sacrificed to measure lung titers. As shown, there were trends toward lower CFU in both VAD and control animals that received VAS at the time of vaccination. A separate set of mice were tested for survival post-challenge. There were significant improvements in survival for VAD and control animals that received VAS at the time of vaccination compared to unsupplemented, vaccinated animals. In fact, all animals that received VAS at the time of vaccination, regardless of original vitamin A status, survived.

## Discussion

We have shown that VAS supports improvements in the immunogenicity of Prevnar-13 in VAD and control mice. With a preliminary study, we also showed that when VAS was given coincident with vaccination, protection against a subsequent challenge with *S. pneumoniae* was improved. Based on these promising results, we are now initiating a randomized clinical study to test the effects of VAS among Memphian children vaccinated with Prevnar-13 (clincaltrials.gov, PCVIT NCT03859687).

### Previous Research on VAD and VAS in Humans

Public health organizations strive to improve dietary nutrition worldwide, but this goal depends on delivering vitamin-rich foods to all populations, a formidable task. For infants in countries where VAD is known to be endemic, the WHO has supported high-dose VAS programs (83). Children often receive 100,000 IU vitamin A between the ages of 6–11 months and 200,000 IU every 4–6 months between the ages of 12–59 months (84). Research analyses of VAS have yielded positive results in countries where VAD is frequent. In meta-analyses of clinical trials, VAS was shown to reduce deaths by 12–24%, and in isolated studies, reductions of 35–50% were observed (1, 85–92). VAS reduced morbidities due to infectious diseases, including measles, *Plasmodium falciparum*, and HIV (93–96). VAS benefits were also observed when antibody responses were measured, including those to vaccination (97, 98). Some studies have shown improved responses to the measles and tetanus toxoid (TT) vaccines following VAS (99–101).

Unfortunately, despite the positive influences described above, results from clinical VAS research have been inconsistent. VAS studies have often failed to show benefit, and have in some cases demonstrated risk. As an example, Malaba et al. did not observe an effect of VAS on infant mortality among children born to HIV-negative mothers with apparently adequate baseline vitamin A levels (102). There have also been reports of increased mother to child transmission (MTCT) of HIV in the context of VAS (103, 104). Additional noted risks of high-dose VAS were fontanelle bulging in infants (105, 106) and bone density loss [possibly due to cross-inhibition between related nuclear hormones, in this case vitamins A and D (107, 108)].

VAS studies in the context of vaccine programs have also yielded conflicting data (97, 109, 110). Brown et al. for example, showed no improvements by VAS on TT vaccinations (110) and a study of HIV-infected individuals showed no improvements by VAS on influenza virus vaccinations (111). A study by Semba et al. showed a negative influence of VAS on responses to the measles vaccine in 6 month old infants (112), unlike the situation for older infants (100, 113, 114). Explanations for differences in VAS efficacy among clinical studies have addressed effects of age, maternal antibodies, serum antibody levels, and serum vitamin levels, but a consensus has not been reached (90).

The contradictory results described above have encouraged the scientific community to question indiscriminate use of VAS, particularly in communities where nutrition has improved and where many children are vitamin replete (97, 106, 115). Suggestions are made to redirect efforts toward the use of low-dose VAS and/or toward support of improved diets (116).

One clear weakness in past clinical research is that comprehensive baseline vitamin levels of study participants for vitamin A and the related, cross-regulatory nuclear hormone vitamin D (47, 117–119) were rarely reported. Instead, vitamin status has often been predicted based on previous population studies (e.g., frequencies of xerophthalmia). This strategy does not address changing diets within communities or individual differences among study participants. Currently, perceptions of VAD frequencies may thus be falsely high for certain developing countries and falsely low for the developed world. The situation differs dramatically from research studies in small animals, wherein host backgrounds and diets are homogeneous and test animals differ from controls by a single defined variable. A full comprehension of how VAS differentially affects humans with replete, insufficient, or deficient vitamin A and D levels remains elusive.

A long-term solution to VAD in humans will require close attention to host characteristics, particularly baseline vitamin A and D levels (52). Improvements in diets should be a primary focus, with VAS programs developed as a back-up solution to malnutrition. For best outcomes with VAS, programs may require customization, with modification of supplements by frequency or dose, dependent on baseline characteristics of vaccine recipients. With attention to pre-existing vitamin levels and cautious administration, VAS programs may ultimately ensure that, (i) vaccinated children and adults are vitamin A replete worldwide, (ii) toxicities are avoided, and (iii) world populations maintain robust immune responses to pathogens and vaccines.

## Data Availability

All datasets generated for this study are included in the manuscript.

## Author Contributions

JH and JR contributed to experimental design, data analyses, and the writing and review of the manuscript. RP, HR, SS, and RS contributed to experimental design, performance of the experiments, data analyses, and the writing and review of the manuscript.

### Conflict of Interest Statement

The authors declare that the research was conducted in the absence of any commercial or financial relationships that could be construed as a potential conflict of interest.

## Abbreviations

RA: retinoic acid
RAR: retinoic acid receptor
RXR: retinoid-X receptor
PPAR: peroxisome proliferator-activated receptor
RARE: retinoic acid response element
VAD: vitamin A deficiency
VAS: vitamin A supplementation
CFU: colony forming units
AID: activation induced deaminase
RDA: recommended daily allowance
RAE: retinoic acid equivalents
RBP: retinol binding protein
WHO: world health organization
ODS: Office of Dietary Supplements
NIH: National Institutes of Health
CSR: class switch recombination
TCR: T cell receptor
IP: intraperitoneally.
